# Supplementary material for: A random forest classifier for protein–protein docking models
Source: Bioinform Adv. 2021 Dec 10;2(1):vbab042. doi: 10.1093/bioadv/vbab042 (PMC9710594; doi:10.1093/bioadv/vbab042)
Supplement: vbab042_Supplementary_Data [file vbab042_supplementary_data.pdf]

Supporting Information of

A Random Forest Classifier for Protein-Protein Docking Models

Didier Barradas-Bautista[1*], Zhen Cao[1], Anna Vangone[2], Romina Oliva[3,*], and Luigi Cavallo[1,*]

[1] Kaust Catalysis Center, Physical Sciences and Engineering Division, King Abdullah University of Science and Technology (KAUST), Thuwal 23955-6900, Saudi Arabia,

[2] Pharma Research and Early Development, Therapeutic Modalities, Roche Innovation Center Munich, LargeMolecule Research, Nonnenwald 2,82377 Penzberg, Germany,

[3] Department of Sciences and Technologies, University Parthenope of Naples, Centro Direzionale Isola C4, I-80143 Naples, Italy.

Table S1. CAPRI criteria for the models assessment, adapted from Lensink et al. (2016) *Proteins*, **84 Suppl 1**, 323-48 (Lensink, et al., 2016).

f(nat)		L-rms (Å)		I-rms (Å)	Assessment
≥ 0.5	AND	≤ 1.0	OR	≤ 1.0	High
≥ 0.3	AND	< 1.0-5.0]	OR	< 1.0-2.0]	Medium
≥ 0.1	AND	< 5.0-10.0]	OR	< 2.0-4.0]	Acceptable
< 0.1	AND	> 10.0	OR	> 4.0	Incorrect

Table S2. Complete list of features used for the initial classifiers training. For each feature, the public source, relative reference and a short description are provided.

Feature	Source	Reference	Description
ACE_COUL	CCharPPI	J. Phys. Chem. 100:1578 (1996) as calculated using CHARMM.	The change in Coulombic energy using the ACE model
ACE_HYDR	CCharPPI	J. Phys. Chem. 100:1578 (1996) as calculated using CHARMM.	The change in hydrophobic energy using the ACE model
ACE_INTE	CCharPPI	J. Phys. Chem. 100:1578 (1996) as calculated using CHARMM.	The change in interaction energy using the ACE model
ACE_SCRE	CCharPPI	J. Phys. Chem. 100:1578 (1996) as calculated using CHARMM.	The change in screening energy using the ACE model
ACE_SELF	CCharPPI	J. Phys. Chem. 100:1578 (1996) as calculated using CHARMM.	The change in self energy using the ACE model
ACE_SOLV	CCharPPI	J. Phys. Chem. 100:1578 (1996) as calculated using CHARMM.	The change in solvation energy (sum of ACE_SCRE and ACE_SELF) using the ACE model
ALIPH	CCharPPI	Proteins 69(1):139 (2007).	The aliphatic potential integrated in FireDock
AP_ACE	CCharPPI	Proteins 69(1):139 (2007).	Contact Energies (J. Mol. Biol. 267(3):707 (1997)) as integrated in FireDock
AP_calRW	CCharPPI	PLoS One. 2010 5(10):e15386.	The calRW distance-dependent atomic potential
AP_calRWp	CCharPPI	PLoS One. 2010 5(10):e15386.	The calRWplus orientation-dependent atomic potential
AP_DARS	CCharPPI	Biophys J. 2008 95(9):4217-27	The DARS potential
AP_DCOMPLEX	CCharPPI	Proteins 56:93 (2004).	The DComplex potential
AP_dDFIRE	CCharPPI	Proteins 72:793 (2008).	Interaction energy calculated using the dDFIRE potential
AP_DDG_U	CCharPPI	J. Chem. Theory Comput. 9(8):3715-3727 (2013).	The unweighted atomic potential derived from mutation data
AP_DDG_W	CCharPPI	J. Chem. Theory Comput. 9(8):3715-3727 (2013).	The weighted atomic potential derived from mutation data

AP_DFIRE2	CCharPPI	Protein Sci. 17:1212 (2008).	Interaction energy calculated using the DFIRE2 potential
AP_DOPE	CCharPPI	Protein Sci. 15(11):2507 (2006).	The DOPE statistical potential
AP_DOPE_HR	CCharPPI	Protein Sci. 15(11):2507 (2006).	The high resolution DOPE statistical potential
AP_GEOMETRIC	CCharPPI	http://gila.bioengr.uic.edu/resources/geometric.html	The unpublished geometric potential of Li X. and Liang J.
AP_GOAP_ALL	CCharPPI	Biophys J. 2011 101(8): 2043-2052.	The total GOAP energy
AP_GOAP_DF	CCharPPI	Biophys J. 2011 101(8): 2043-2052.	The DFIRE term in the GOAP energy
AP_GOAP_G	CCharPPI	Biophys J. 2011 101(8): 2043-2052.	The GOAP_ag term in the GOAP energy
AP_MPS	CCharPPI	Biophys J. 2008 95(9):4217-27	The MPS potential
AP_OPUS_PSP	CCharPPI	J. Mol. Biol. 376:288 (2008).	The OPUS-PSP potential
AP_PISA	CCharPPI	Proteins 81(4):592 (2013).	The PISA score
AP_T1	CCharPPI	BMC Struct biol 10:40 (2010).	The first atomic two-step potential
AP_T2	CCharPPI	BMC Struct biol 10:40 (2010).	The second atomic two-step potential
AP_URS	CCharPPI	Biophys J. 2008 95(9):4217-27	The URS potential
AP_W1	CCharPPI	Proteins 2007 69(3):511-20.	A reimplementation of the potential
CP_BFKV	CCharPPI	Proteins 59(1):49 (2005) and BMC Bioinformatics 11:92 (2010)	Contact potenital calculated between intermolecular residues.
CP_BL	CCharPPI	Proteins 59(1):49 (2005) and BMC Bioinformatics 11:92 (2010)	Contact potenital calculated between intermolecular residues.
CP_BT	CCharPPI	Proteins 59(1):49 (2005) and BMC Bioinformatics 11:92 (2010)	Contact potenital calculated between intermolecular residues.
CP_D1	CCharPPI	BMC Bioinformatics. 2011 12:280.	A reimplementation of the DECK residue level distance-dependent potential
CP_DDG_U	CCharPPI	J. Chem. Theory Comput. 9(8):3715-3727 (2013).	The unweighted residue potential derived from mutation data
CP_DDG_W	CCharPPI	J. Chem. Theory Comput. 9(8):3715-3727 (2013).	The weighted residue potential derived from mutation data
CP_E3D_CB	CCharPPI	Protein Sci. 2011	The E_3D C_beta potential

		20(3):529-41.	
CP_E3D_MIN	CCharPPI	Protein Sci. 2011 20(3):529-41.	The E_3D R_min potential
CP_E3DC_CB	CCharPPI	Protein Sci. 2011 20(3):529-41.	The E_3DC C_beta potential
CP_E3DC_MIN	CCharPPI	Protein Sci. 2011 20(3):529-41.	The E_3DC R_min potential
CP_ELOCAL_CB	CCharPPI	Protein Sci. 2011 20(3):529-41.	The E_local C_beta potential
CP_ELOCAL_MIN	CCharPPI	Protein Sci. 2011 20(3):529-41.	The E_local R_min potential
CP_EPAIR_CB	CCharPPI	Protein Sci. 2011 20(3):529-41.	The E_pair C_beta potential
CP_EPAIR_MIN	CCharPPI	Protein Sci. 2011 20(3):529-41.	The E_pair R_min potential
CP_ES3DC_CB	CCharPPI	Protein Sci. 2011 20(3):529-41.	The E_ZS3DC C_beta potential
CP_ES3DC_MIN	CCharPPI	Protein Sci. 2011 20(3):529-41.	The E_ZS3DC R_min potential
CP_GKS	CCharPPI	Proteins 59(1):49 (2005) and BMC Bioinformatics 11:92 (2010)	Contact potenital calculated between intermolecular residues.
CP_HLPL	CCharPPI	Proteins 59(1):49 (2005) and BMC Bioinformatics 11:92 (2010)	Contact potenital calculated between intermolecular residues.
CP_MJ1	CCharPPI	Proteins 59(1):49 (2005) and BMC Bioinformatics 11:92 (2010)	Contact potenital calculated between intermolecular residues.
CP_MJ2	CCharPPI	Proteins 59(1):49 (2005) and BMC Bioinformatics 11:92 (2010)	Contact potenital calculated between intermolecular residues.
CP_MJ2h	CCharPPI	Proteins 59(1):49 (2005) and BMC Bioinformatics 11:92 (2010)	Contact potenital calculated between intermolecular residues.
CP_MJ3h	CCharPPI	Proteins 59(1):49 (2005) and BMC Bioinformatics 11:92 (2010)	Contact potenital calculated between intermolecular residues.
CP_MJPL	CCharPPI	Proteins 59(1):49 (2005) and BMC Bioinformatics 11:92 (2010)	Contact potenital calculated between intermolecular residues.
CP_MS	CCharPPI	Proteins 59(1):49 (2005) and BMC Bioinformatics 11:92 (2010)	Contact potenital calculated between intermolecular residues.
CP_MSBM	CCharPPI	Proteins 59(1):49 (2005) and BMC Bioinformatics 11:92	Contact potenital calculated between intermolecular residues.

		(2010)	
CP_Qa	CCharPPI	Proteins 59(1):49 (2005) and BMC Bioinformatics 11:92 (2010)	Contact potenital calculated between intermolecular residues.
CP_Qm	CCharPPI	Proteins 59(1):49 (2005) and BMC Bioinformatics 11:92 (2010)	Contact potenital calculated between intermolecular residues.
CP_Qp	CCharPPI	Proteins 59(1):49 (2005) and BMC Bioinformatics 11:92 (2010)	Contact potenital calculated between intermolecular residues.
CP_RMFCA	CCharPPI	Proteins 65(3):726 (2006).	The C_alpha-C_alpha potential
CP_RMFCEN1	CCharPPI	Proteins 70(3):950 (2006).	The 6bin-HRSC centroid-centroid potential
CP_RMFCEN2	CCharPPI	Proteins 70(3):950 (2006).	The 7bin-HRSC centroid-centroid potential
CP_RO	CCharPPI	Proteins 59(1):49 (2005) and BMC Bioinformatics 11:92 (2010)	Contact potenital calculated between intermolecular residues.
CP_SJKG	CCharPPI	Proteins 59(1):49 (2005) and BMC Bioinformatics 11:92 (2010)	Contact potenital calculated between intermolecular residues.
CP_SKOa	CCharPPI	Proteins 59(1):49 (2005) and BMC Bioinformatics 11:92 (2010)	Contact potenital calculated between intermolecular residues.
CP_SKOb	CCharPPI	Proteins 59(1):49 (2005) and BMC Bioinformatics 11:92 (2010)	Contact potenital calculated between intermolecular residues.
CP_SKOIP	CCharPPI	Biophys. J. 84(3):1895 (2003).	The residue level interaction contact potential
CP_TB	CCharPPI	Proteins 62(4):970 (2006).	The residue level interaction contact potential
CP_TD	CCharPPI	Proteins 59(1):49 (2005) and BMC Bioinformatics 11:92 (2010)	Contact potenital calculated between intermolecular residues.
CP_TEl	CCharPPI	Proteins 59(1):49 (2005) and BMC Bioinformatics 11:92 (2010)	Contact potenital calculated between intermolecular residues.
CP_TEs	CCharPPI	Proteins 59(1):49 (2005) and BMC Bioinformatics 11:92 (2010)	Contact potenital calculated between intermolecular residues.
CP_TS	CCharPPI	Proteins 59(1):49 (2005) and BMC Bioinformatics 11:92	Contact potenital calculated between intermolecular residues.

		(2010)	
CP_TSC	CCharPPI	BMC Struct biol 10:40 (2010).	The residue level interaction two-step potential
CP_VD	CCharPPI	Proteins 59(1):49 (2005) and BMC Bioinformatics 11:92 (2010)	Contact potenital calculated between intermolecular residues.
CP_Z3DC_CB	CCharPPI	Protein Sci. 2011 20(3):529-41.	The E_3DC Z-score C_beta potential
CP_Z3DC_MIN	CCharPPI	Protein Sci. 2011 20(3):529-41.	The E_3DC Z-score R_min potential
CP_ZLOCAL_CB	CCharPPI	Protein Sci. 2011 20(3):529-41.	The E_local Z-score C_beta potential
CP_ZLOCAL_MIN	CCharPPI	Protein Sci. 2011 20(3):529-41.	The E_local Z-score R_min potential
CP_ZPAIR_CB	CCharPPI	Protein Sci. 2011 20(3):529-41.	The E_pair Z-score C_beta potential
CP_ZPAIR_MIN	CCharPPI	Protein Sci. 2011 20(3):529-41.	The E_pair Z-score R_min potential
CP_ZS3DC_CB	CCharPPI	Protein Sci. 2011 20(3):529-41.	The E_ZS3DC z-score C_beta potential
CP_ZS3DC_MIN	CCharPPI	Protein Sci. 2011 20(3):529-41.	The E_ZS3DC z-score R_min potential
DDG_V	CCharPPI	Proteins 83(4):640 (2015).	A microscopic surface energy model derived from mutation data
DESOLV	CCharPPI	Proteins 68:503 (2007) and Protein 69:852 (2007).	Desolvation energy as calculated using pyDock
DOKB	CCharPPI	Biopolymers 101(6):681 (2014).	Energy is calculated by summating the interaction energy between residues which is generated by Boltzmann statistic. DOI:10.1002/bip.22440
ELE	CCharPPI	Proteins 68:503 (2007) and Protein 69:852 (2007).	Total electrostatic energy as calculated using PyDock
FIREDOCK	CCharPPI	Proteins 69(1):139 (2007).	The total FireDock energy (default energy function)
FIREDOCK_AB	CCharPPI	Proteins 69(1):139 (2007).	The total FireDock energy (antibody-antigen energy function)
FIREDOCK_EI	CCharPPI	Proteins 69(1):139 (2007).	The total FireDock energy (enzyme-inhibitor energy function)
HBOND	CCharPPI	Proteins 69(1):139 (2007).	The hydrogen bonding potential integrated in FireDock
INSIDE	CCharPPI	Proteins 69(1):139 (2007).	Insideness concavity as integrated in FireDock
NSC	CCharPPI	FEBS Lett. 584(6):1163 (2010).	The surface complementarity score

ODA	CCharPPI	Int. J. Data Mining Bioinf. 3:55 (2009) and J. Chem. Inf. Mod. 51:370 (2011).	The optimal docking area (ODA) score
PI_PI	CCharPPI	Proteins 69(1):139 (2007).	The pi-pi potential integrated in FireDock
PROPNSTS	CCharPPI	J. Chem. Inf. Mod. 51:370 (2011).	Amino acid propensity score
PYDOCK_TOT	CCharPPI	Proteins 68:503 (2007) and Protein 69:852 (2007).	Total pyDock energy Proteins 68:503 (2007) and Protein 69:852 (2007).
ROT_S	CCharPPI		Change in rotational entropy upon complexation as calculated using CHARMM.
SASA	CCharPPI	Proteins 46:24 (2002)	The SASA implicit solvation model as calculated using CHARMM.
SIPPER	CCharPPI	J. Chem. Inf. Mod. 51:370 (2011).	The SIPPER potential
TRANS_S	CCharPPI		Change in translational entropy upon complexation as calculated using CHARMM
VDW	CCharPPI	Proteins 68:503 (2007) and Proteins 69:852 (2007).	Van der Waals energy as calculated using pyDock
ZRANK	CCharPPI	Proteins 67:1078 (2007)	The ZRANK scoring function
ZRANK2	CCharPPI	Proteins 72:270 (2008)	The ZRANK2 scoring function
avg_cips_AA	CIPS	Bioinformatics 34:459 (2018)	Sum of CIPS score for Apolar-Apolar contacts over the total number of Apolar-Apolar contacts
avg_cips_AAl	CIPS	Bioinformatics 34:459 (2018)	Sum of CIPS score for Apolar-Aliphatic contacts over the total number of Apolar-Aliphatic contacts
avg_cips_AAr	CIPS	Bioinformatics 34:459 (2018)	Sum of CIPS score for Apolar-Aromatic contacts over the total number of Apolar-Aromatic contacts
avg_cips_AlAl	CIPS	Bioinformatics 34:459 (2018)	Sum of CIPS score for Aliphatic-Aliphatic contacts over the total number of Aliphatic-Aliphatic contacts
avg_cips_AlAr	CIPS	Bioinformatics 34:459 (2018)	Sum of CIPS score for Aliphatic-Aromatic contacts over the total number of Aliphatic-Aromatic contacts

avg_cips_ArAr	CIPS	Bioinformatics 34:459 (2018)	Sum of CIPS score for Aromatic-Aromatic contacts over the total number of Aromatic-Aromatic contacts
avg_cips_CA	CIPS	Bioinformatics 34:459 (2018)	Sum of CIPS score for Charged-Apolar contacts over the total number of Charged-Apolar contacts
avg_cips_CAl	CIPS	Bioinformatics 34:459 (2018)	Sum of CIPS score for Charged-Aliphatic contacts over the total number of Charged-Aliphatic contacts
avg_cips_CAr	CIPS	Bioinformatics 34:459 (2018)	Sum of CIPS score for Charged-Aromatic contacts over the total number of Charged-Aromatic contacts
avg_cips_CC	CIPS	Bioinformatics 34:459 (2018)	Sum of CIPS score for Charged-Charged contacts over the total number of Charged-Charged contacts
avg_cips_CP	CIPS	Bioinformatics 34:459 (2018)	Sum of CIPS score for Charged-Polar contacts over the total number of Charged-Polar contacts
avg_cips_PAl	CIPS	Bioinformatics 34:459 (2018)	Sum of CIPS score for Polar-Aliphatic contacts over the total number of Polar-Aliphatic contacts
avg_cips_PAr	CIPS	Bioinformatics 34:459 (2018)	Sum of CIPS score for Polar-Aromatic contacts over the total number of Polar-Aromatic contacts
avg_cips_PP	CIPS	Bioinformatics 34:459 (2018)	Sum of CIPS score for Polar-Polar contacts over the total number of Polar-Polar contacts
cips_AA	CIPS	Bioinformatics 34:459 (2018)	Sum of CIPS score for Apolar-Apolar contacts
cips_AAl	CIPS	Bioinformatics 34:459 (2018)	Sum of CIPS score for Apolar-Aliphatic contacts
cips_AAr	CIPS	Bioinformatics 34:459 (2018)	Sum of CIPS score for Apolar-Aromatic contacts
cips_AlAl	CIPS	Bioinformatics 34:459 (2018)	Sum of CIPS score for Aliphatic-Aliphatic contacts
cips_AlAr	CIPS	Bioinformatics 34:459 (2018)	Sum of CIPS score for Aliphatic-Aromatic contacts
cips_ArAr	CIPS	Bioinformatics 34:459 (2018)	Sum of CIPS score for Aromatic-Aromatic contacts
cips_CA	CIPS	Bioinformatics 34:459 (2018)	Sum of CIPS score for Charged-Apolar contacts

cips_CAl	CIPS	Bioinformatics 34:459 (2018)	Sum of CIPS score for Charged-Alphatic contacts
cips_CC	CIPS	Bioinformatics 34:459 (2018)	Sum of CIPS score for Charged-Charged contacts
cips_CP	CIPS	Bioinformatics 34:459 (2018)	Sum of CIPS score for Charged-Polar contacts
cips_PA	CIPS	Bioinformatics 34:459 (2018)	Sum of CIPS score for Polar-Apolar contacts
cips_PAl	CIPS	Bioinformatics 34:459 (2018)	Sum of CIPS score for Polar-Aliphatic contacts
cips_PP	CIPS	Bioinformatics 34:459 (2018)	Sum of CIPS score for Polar-Polar contacts
avg_cips_PA	CIPS	Bioinformatics 34:459 (2018)	Sum of CIPS score for Polar-Apolar contacts over the total number of Polar-Apolar contacts
CONSRANK score	CONSRANK	Bioinformatics 31:1481 (2015) Proteins 81:1571 (2013)	CONSRANK normalized score, reflecting the conservation of the inter-residue contacts of a given model in the decoys set
num_of_contacts	CONSRANK	Bioinformatics 31:1481 (2015) Proteins 81:1571 (2013)	Total number of contacts as calculated in ConsRank
AA	COCOMAPS	Bioinformatics 27:2915 (2011)	Apolar-Apolar contact count at 5 Å distance
AA_sqrt	COCOMAPS	Bioinformatics 27:2915 (2011)	Square Root of Apolar-Apolar contact count at 5 Å distance
AAl	COCOMAPS	Bioinformatics 27:2915 (2011)	Apolar-Aliphatic contact count at 5 Å distance
AAl_sqrt	COCOMAPS	Bioinformatics 27:2915 (2011)	Square Root of Apolar-Aliphatic contact count at 5 Å distance
AAr	COCOMAPS	Bioinformatics 27:2915 (2011)	Apolar-Aromatic contact count at 5 Å distance
AAr_sqrt	COCOMAPS	Bioinformatics 27:2915 (2011)	Square Root of Apolar-Aromatic contact count at 5 Å distance
AlAl	COCOMAPS	Bioinformatics 27:2915 (2011)	Aliphatic-Aliphatic contact count at 5 Å distance
AlAl_sqrt	COCOMAPS	Bioinformatics 27:2915 (2011)	Square Root of Aliphatic-Aliphatic contact count at 5 Å distance
AlAr	COCOMAPS	Bioinformatics 27:2915 (2011)	Aliphatic-Aromatic contact count at 5 Å distance
AlAr_sqrt	COCOMAPS	Bioinformatics 27:2915 (2011)	Square Root of Aliphatic-Aromatic contact count at 5 Å distance
ArAr	COCOMAPS	Bioinformatics 27:2915 (2011)	Aromatic-Aromatic contact count at 5 Å distance
ArAr_sqrt	COCOMAPS	Bioinformatics 27:2915 (2011)	Square Root of Aromatic-Aromatic contact count at 5

			Å distance
CA	COCOMAPS	Bioinformatics 27:2915 (2011)	Charged-Apolar contact count at 5 Å distance
CA_sqrt	COCOMAPS	Bioinformatics 27:2915 (2011)	Square Root of Charged-Apolar contact count at 5 Å distance
CAl	COCOMAPS	Bioinformatics 27:2915 (2011)	Charged-Aliphatic contact count at 5 Å distance
CAl_sqrt	COCOMAPS	Bioinformatics 27:2915 (2011)	Square Root of Charged-Aliphatic contact count at 5 Å distance
CAr	COCOMAPS	Bioinformatics 27:2915 (2011)	Charged-Aromatic contact count at 5 Å distance
CAr_sqrt	COCOMAPS	Bioinformatics 27:2915 (2011)	Square Root of Charged-Aromatic contact count at 5 Å distance
CC	COCOMAPS	Bioinformatics 27:2915 (2011)	Charged-Charged contact count at 5 Å distance
CC_sqrt	COCOMAPS	Bioinformatics 27:2915 (2011)	Square Root of Charged-Charged contact count at 5 Å distance
CP	COCOMAPS	Bioinformatics 27:2915 (2011)	Charged-Polar contact count at 5 Å distance
CP_sqrt	COCOMAPS	Bioinformatics 27:2915 (2011)	Square Root of Charged-Polar contact count at 5 Å distance
PA	COCOMAPS	Bioinformatics 27:2915 (2011)	Polar-Apolar contact count at 5 Å distance
PA_sqrt	COCOMAPS	Bioinformatics 27:2915 (2011)	Square Root of Polar-Apolar contact count at 5 Å distance
PAl	COCOMAPS	Bioinformatics 27:2915 (2011)	Polar-Aliphatic contact count at 5 Å distance
PAl_sqrt	COCOMAPS	Bioinformatics 27:2915 (2011)	Square Root of Polar-Aliphatic contact count at 5 Å distance
PAr	COCOMAPS	Bioinformatics 27:2915 (2011)	Polar-Aromatic contact count at 5 Å distance
PAr_sqrt	COCOMAPS	Bioinformatics 27:2915 (2011)	Square Root of Polar-Aromatic contact count at 5 Å distance
PP	COCOMAPS	Bioinformatics 27:2915 (2011)	Polar-Polar contact count at 5 Å distance
PP_sqrt	COCOMAPS	Bioinformatics 27:2915 (2011)	Square Root of Polar-Polar contact count at 5 Å distance
BSA	Freesasa	F1000Research 5:189 (2016)	Total Buried Surface Area from FreeSASA
BSA_Apolar	Freesasa	F1000Research 5:189 (2016)	Polar Buried Surface Area from FreeSASA
BSA_Polar	Freesasa	F1000Research 5:189 (2016)	Apolar Buried Surface Area from FreeSASA
Nis_Apolar	Prodigy	eLife 4: 291 (2015)	Percentage of charged non-

		J Mol Biol 426: 2632 (2014)	interacting surface (NIS) on residues from Binding affinity predictor based on Intermolecular Contacts (ICs).
Nis_Polar	Prodigy	eLife 4: 291 (2015) J Mol Biol 426: 2632 (2014)	Percentage of apolar non-interacting surface (NIS) on residues from Binding affinity predictor based on Intermolecular Contacts (ICs).

Table S3. Global parameters: Accuracy (Acc), Recall (R), Precision (P), F1_score (F1), Matthews' correlation coefficient (MCC) for all the discussed classifiers. These values are also reported in the form of radar plots in the main text.

Classifier	Acc	R_inc	R_cor	P_inc	P_corr	F1_inc	F1_cor	MCC	Validation set	Figs
RF	0.8321	0.8669	0.7973	0.8105	0.8569	0.8377	0.8260	0.6658	Bal-BM5-up	2,3
PRC	0.8184	0.8274	0.8094	0.8128	0.8242	0.8200	0.8168	0.6369	Bal-BM5-up	2
SVM	0.7170	0.6863	0.7477	0.7312	0.7045	0.7080	0.7254	0.4348	Bal-BM5-up	2
RF	0.9164	0.9232	0.7985	0.9874	0.3777	0.9543	0.5128	0.5133	3K-BM5-up	2,3,5
PRC	0.8683	0.8726	0.7957	0.9865	0.2670	0.9261	0.3998	0.4116	3K-BM5-up	2
SVM	0.7085	0.7072	0.7299	0.9782	0.1270	0.8209	0.2163	0.2144	3K-BM5-up	2
RF	0.8222	0.9246	0.7199	0.768	0.9058	0.8388	0.8017	0.659	Bal-10CV-BM5	3
RF	0.9511	0.9624	0.7341	0.9859	0.5043	0.974	0.5973	0.584	3K-BM5-up	3
CoDES	0.9183	0.9197	0.8989	0.9916	0.4618	0.9543	0.6101	0.6093	3K-BM5-up	5
CoDES	0.7073	0.6712	0.9852	0.9971	0.2803	0.8023	0.4364	0.4267	Score_set	5
CoDES	0.6937	0.6216	0.9948	0.9980	0.3864	0.7661	0.5566	0.4867	Score_set (>2.5%)	5

Table S4. Global parameters: Accuracy (Acc), Recall (R), Precision (P), F1_score (F1), Matthews' correlation coefficient (MCC) for five tree-based classifiers on the 3K-BM5-up validation set. For each metric, the maximum value per metric among the five clssifiers is highlighted in bold.

Classifier	Acc	R_inc	R_corr	P_inc	P_corr	F1_inc	F1_corr	MCC
RF	**0.9164**	**0.9232**	0.7985	0.9874	**0.3777**	**0.9543**	0.5128	0.5133
XgBoost	0.8867	0.8862	0.8937	0.9909	0.3756	0.9356	**0.5290**	**0.5347**
Gradient boosting	0.8632	0.8572	0.9417	0.9948	0.3357	0.9209	0.4949	0.5138
Decision Tree	0.8489	0.8418	0.9423	0.9948	0.3133	0.9119	0.4702	0.4915
AdaBoost	0.8415	0.8334	**0.9472**	**0.9952**	0.3034	0.9071	0.4596	0.4828

Table S5. Features with the highest importance are reported for the RF classifier trained on the balanced sets with a BM4/BM5-update and 10-fold cross-validation approach. Features are sorted based on values of the second column. The mean importance value and associated standard deviation is reported in the last column. The 16 features selected because having an importance above 0.01 for both the classifiers were selected – they are reported in bold. Values below the importance threshold of 0.01 and the corresponding features are reported in italics.

Feature	Bal-BM4/5-up	Bal-10CV-BM5	CoDES (rank)
**CONSRANK_score**	0.2056	0.1978+/-0.0074	0.3257 (1)
**AP_GOAP_DF**	0.0275	0.0168+/-0.0027	0.0615 (5)
**CP_TD**	0.0247	0.0136+/-0.0013	0.0305 (15)
**CP_D1**	0.0240	0.0151+/-0.0025	0.0694 (2)
**CP_HLPL**	0.0225	0.0256+/-0.0018	0.0388 (11)
**DDG_V**	0.0225	0.0226+/-0.0021	0.0508 (6)
**CP_MJ3h**	0.0224	0.0229+/-0.0031	0.0376 (12)
**pyDock_TOT**	0.0223	0.0232+/-0.0022	0.0463 (7)
**ELE**	0.0177	0.0192+/-0.0013	0.0682 (3)
**CP_SKOIP**	0.0172	0.0233+/-0.0039	0.0374 (13)
CP_MJ2h	0.0170	*0.0089+/-0.0023*	-
**SIPPER**	0.0149	0.0171+/-0.0029	0.0402 (10)
**AP_DFIRE2**	0.0129	0.0113+/-0.0018	0.0562 (4)
**AP_dDFIRE**	0.0120	0.0127+/-0.0026	0.0458 (8)
**AP_PISA**	0.0118	0.0138+/-0.0025	0.0412 (9)
**CP_RMFCA**	0.0101	0.0129+/-0.0015	0.0346 (14)
**CP_TSC**	0.0101	0.0115+/-0.0014	0.0160 (16)
CP_TB	*0.0093*	0.0141+/-0.0024	-

CP_BT	*0.0083*	0.0104+/-0.0029	-
AP_DARS	*0.0075*	0.0105+/-0.0018	-

Table S6. Features with the top ten importance are reported for the final CoDES classifier in comparison with the importance of the same features in the preliminary RF classifier trained on the balanced sets with a BM4/BM5-update validation approach. Features are sorted based on values of the second column.

Feature	CoDES	Bal-BM4/5-up (rank)
CONSRANK_score	0.3257	0.2056 (1)
CP_D1	0.0694	0.0240 (4)
ELE	0.0682	0.0177 (9)
AP_GOAP_DF	0.0615	0.0275 (2)
AP_DFIRE2	0.0562	0.0129 (13)
DDG_V	0.0508	0.0225 (6)
pyDock_TOT	0.0463	0.0223 (8)
AP_PISA	0.0462	0.0118 (15)
AP_dDFIRE	0.0458	0.0120 (14)
CP_RMFCA	0.0406	0.0120 (16)
